# Climate change conditions the selection of rust-resistant candidate wild lentil populations for *in situ* conservation

**DOI:** 10.3389/fpls.2022.1010799

**Published:** 2022-11-03

**Authors:** Iciar Civantos-Gómez, María Luisa Rubio Teso, Javier Galeano, Diego Rubiales, José María Iriondo, Javier García-Algarra

**Affiliations:** ^1^ Complex System Group, Universidad Politécnica de Madrid, Madrid, Spain; ^2^ Faculty of Economics and Business Administration, Universidad Pontificia Comillas, Madrid, Spain; ^3^ ECOEVO Research Group, Área de Biodiversidad y Conservación, Universidad Rey Juan Carlos, Madrid, Spain; ^4^ Instituto de Agricultura Sostenible (CSIC) Avenida Menéndez Pidal s/n Campus Alameda del Obispo, Córdoba, Spain; ^5^ DRACO Research Group, Centro Universitario de Tecnología y Arte Digital, Las Rozas, Spain

**Keywords:** crop wild relatives, climate change, machine learning, rust resistance, lentils, *in situ* conservation, predictive characterization

## Abstract

Crop Wild Relatives (CWR) are a valuable source of genetic diversity that can be transferred to commercial crops, so their conservation will become a priority in the face of climate change. Bizarrely, *in situ* conserved CWR populations and the traits one might wish to preserve in them are themselves vulnerable to climate change. In this study, we used a quantitative machine learning predictive approach to project the resistance of CWR populations of lentils to a common disease, lentil rust, caused by fungus *Uromyces viciae-fabae*. Resistance is measured through a proxy quantitative value, DSr (Disease Severity relative), quite complex and expensive to get. Therefore, machine learning is a convenient tool to predict this magnitude using a well-curated georeferenced calibration set. Previous works have provided a binary outcome (resistant *vs.* non-resistant), but that approach is not fine enough to answer three practical questions: which variables are key to predict rust resistance, which CWR populations are resistant to rust under current environmental conditions, and which of them are likely to keep this trait under different climate change scenarios. We first predict rust resistance in present time for crop wild relatives that grow up inside protected areas. Then, we use the same models under future climate IPCC (Intergovernmental Panel on Climate Change) scenarios to predict future DSr values. Populations that are rust-resistant by now and under future conditions are optimal candidates for further evaluation and *in situ* conservation of this valuable trait. We have found that rust-resistance variation as a result of climate change is not uniform across the geographic scope of the study (the Mediterranean basin), and that candidate populations share some interesting common environmental conditions.

## 1 Introduction

In the coming decades, food security will be seriously compromised by the lack of adaptive resilience to climate change of cultivars currently used in crops (Smith et al., 2017; Wiebe et al., 2019; Anderson et al., 2020). This lack of adaptive resilience is caused by the low genetic diversity that is inherent to most modern cultivars (Rauf et al., 2010; Van de Wouw et al., 2010; Rufo et al., 2019). Indeed, it has been estimated that major crops are likely to experience sensible yield reductions in the coming decades. In their review of 2015 for Eastern Africa, Adhikari et al. depict a gloomy scenario for main staples by the end of this century. They predict a 72% drop for wheat, around 40% for other cereals, and 10% for potatoes (Adhikari et al., 2015). A recent study predicts a drop that ranges from 3% to 12% by 2050 and from 11% to 25% by 2090 for rice and soybeans (Wing et al., 2021). This will be the result of losses caused by the arrival of new pests and pathogens, the intensification of the effects of those active right now, and potential mismatches to the new climate regimes, including increasing temperature and drought and higher incidence of extreme events (e.g., hail, strong winds, floods, etc). Anyway, those statistical projections could hide the fact that climate change may result beneficial for some crops and regions (Ray et al., 2019).

Crop wild relatives (CWR) are one of the most important sources of genetic diversity to transfer key adaptations to crops, a relevant fact in the context of climate change (Heywood et al., 2007; Maxted, 2008; Zhang et al., 2017). For example, they have been effectively used in plant breeding in crops such as sunflower (*Helianthus annuus* L.) (Seiler et al., 2017), narrow−leafed lupin (*Lupinus angustifolius* L.) (Mousavi-Derazmahalleh et al., 2018), durum wheat (*Triticum turgidum* subsp. *durum* (Desf.) Husn.) (El Haddad et al., 2021) or pea (*Pisum sativum* L.) (Rubiales et al., 2020).

Interestingly, the genetic diversity of crop wild relatives is, at the same time, being severely eroded mainly due to habitat modification and destruction by human activities (Iriondo et al., 2008; Khoury et al., 2022). A recent survey of 600 species of crop wild relatives in the United States, estimated that more than one-half are endangered and 7% in a critical condition (Khoury et al., 2020). As a result, a global effort is being made to promote the establishment of genetic reserves or the *in situ* conservation of crop wild relatives (Maxted et al., 2008; van Treuren et al., 2017; Labokas et al., 2018).

In the process of deciding which populations of a given crop wild relative should be selected for *in situ* conservation, there are a number of considerations that must be taken into account. On one hand, genetic reserves should be representative of the range of genetic diversity present in the species (Dempewolf et al., 2017). The best way of characterizing genetic diversity is through sequencing as it provides complete information about the genome and its cost is drastically decreasing. Nevertheless, the characterization of hundreds or thousands of populations that a given crop wild relative might have is a highly time-consuming task that would require huge human and economic resources as well. Ecogeographic information arises as a useful tool that provides a proxy to estimate among-population genetic diversity and helps in the selection of representative populations (Vincent et al., 2019). Plant breeders are often interested in identifying and conserving candidates that display a targeted phenotypic trait (e.g., resistance to a pathogen). Once again, ecogeographic information associated with the CWR populations is critical to identifying these candidate populations given the impossibility of conducting evaluation experiments with plant material from so many sites. In this sense, predictive characterization techniques based on FIGS (Focused Identification Germplasm Strategy) have been applied to identify wheat resistance to stem rust, caused by the fungus *Puccinia graminis* (Endresen et al., 2012; Bari et al., 2014), and barley resistance to leaf rust, caused by *Puccinia hordei* (Amouzoune et al., 2022). Different methodological approaches predicted phenotypic traits through a calibration approach, using as a starting point a set of training data where the targeted trait of a set of populations is known and the corresponding ecogeographic information may be retrieved from public repositories (Endresen, 2010; Sánchez et al., 2019). In the search for resistance to pathogens, several researchers have followed qualitative approaches in which the material is previously evaluated as resistant or non-resistant (Bari et al., 2012; Rubio Teso et al., 2022). However, resistance to pathogens could be evaluated using numerical variables as well (Arojju et al., 2018; Ren et al., 2021). Predictive characterization of quantitative resistance traits such as DSr (Disease Severity relative) to lentil rust (*Uromyces vicia-fabae* (Pers.) Schröt), is likely to benefit from machine-learning models with quantitative dependent variables (Rubiales et al., 2013).

The second aspect of great relevance when identifying the most appropriate CWR populations relates to the adequacy of the site for the long term *in situ* conservation. The land use of the site has to be compatible with the long-term viability and persistence of the candidate population (Hunter, 2012; Hunter et al., 2012). Those within protected areas are less vulnerable to human disturbance and are, therefore, preferred in this context (Maxted et al., 2012). In any case, the *in situ* conservation of CWR in genetic reserves may also be feasible in other instances (e.g., farms), whenever there is a long-term commitment by the landowners (Maxted and Kell, 2009).

*Ex situ* conservation is the best and most adequate approach for conserving and utilizing plant genetic reserves. One of the main benefits of complementing it with *in situ* conservatio of CWR is that *in situ* conserved wild populations are constantly evolving as a result of changing biotic and abiotic environmental factors (Fu, 2017). Adapting to such changes favours genotypes that maximize their fitness under current and potentially future environmental conditions (Meilleur and Hodgkin, 2004). On the contrary, the germplasm conserved ex situ in genebanks allows rapid access to genetic variation but represent a static genetic diversity of the population in the moment of sampling (Castañeda-Álvarez et al., 2016) that, in any case can be useful in recovering populations. In the identification of the most appropriate CWR populations for *in situ* conservation, one must take into account not only the environmental conditions currently present in the target population but also those expected to occur in the future as a result of climate change, and whether those future conditions are compatible with the preservation of the population or the targeted traits. The vulnerability of protected areas to the effects of climate change is starting to be assessed in terms of global biodiversity or emblematic species but has not been studied in the context of CWR *in situ* conservation (Hannah, 2010; Triviño et al., 2018).

In this study we used a predictive characterization approach, based on machine learning, to quantitatively project the rust resistance of crop wild relative populations of lentils (*Lens culinaris* subsp. *culinaris*) in the Mediterranean basin. Rust is a severe foliar disease in lentils (Rubiales et al., 2011). Breeding with CWRs to increase rust resistance of cultivars is a convenient method for this disease control in legume crops (Barilli et al., 2009; Rubiales et al., 2011; Negussie and Pretorius, 2012; Sillero et al., 2017).

Climate change is expected to unevenly affect agriculture in different parts of the world (Howden et al., 2007). There is large variation in climatic conditions, soils, land use, infrastructure, and political and economic conditions across the European continent (Olesen and Bindi, 2002; Cramer et al., 2018; Fellmann et al., 2018). These differences are expected to influence the responsiveness of CWR populations to climate change. Here we apply climate change projections and Shared Socio-Economic Pathways (SSPs) to predictive characterization in order to compare the potential changes in rust-resistance of lentil CWR populations in Europe and Turkey (Dufresne et al., 2013; Voldoire et al., 2013; O’Neill et al., 2014; Wu et al., 2014). We, then, aimed to identify a set of rust-resistant candidate populations that could be designated for *in situ* conservation in genetic reserves, searching for those that occur in protected areas and selecting those with the lowest vulnerability to changes in the environment that might result in the loss of this trait. The results of the calibration method of the predictive characterization techniques should answer the following questions: (i) which variables are the most important to predict rust resistance? (ii) which CWR populations are likely to show strong resistance to rust under present time environmental conditions? and (iii) which of them are likely to keep this trait under forthcoming climate change scenarios? We expect selected populations to have evolved to develop rust resistance over time and consider them to be a valuable asset under present and future conditions.

## 2 Data description

Lentil was cultivated for the first time in the Fertile Crescent around 5000 BP (Zohary et al., 2012). It probably spread out through the Mediterranean basin, the Indian subcontinent, and the Horn of Africa at a relatively fast pace driven by its high yield (Liber et al., 2021). Thus, it didn’t face the selective pressure of its wild relatives, the subspecies *orientalis* and *odemensis* and the three species *Lens nigricans* (M. Bieb.) Godr., *Lens ervoides* (Brign.) Grande and *Lens lammotei*. Czefr. We considered in our study all *Lens* taxa naturally occurring in Europe and Turkey. These are *L. ervoides*, *L. nigricans* and *L. lammotei*, as well as *L. culinaris* subsp. *orientalis* (Boiss.) Ponert. and *L. culinaris* subsp. *odemensis*. (Ladiz.). *Lens* taxa distribution data were extracted from a database of crop wild relative populations in Europe and Turkey generated for the Farmer’s Pride project (www.farmerspride.eu) (Rubio Teso et al., 2020). Due to the imbalance in the number of samples ofthe original database (443 populations of *L. nigricans* found in 12 countries, 145 of *L. ervoides* in 9 countries, 29 of *L. lamottei* and 9 of *L. culinaris subsp. orientalis*), we decided to build a unique model and exclude the species as an input variable. We made this choice to avoid overfitted models, being aware that there is a loss of input information, but taking into account that the four taxa belong to the same genus.

Raw data downloaded were further cleaned and filtered as indicated in (Rubio Teso et al., 2022). The calibration dataset holding rust evaluation data has 351 samples of five *Lens* taxa, both wild and cultivated (*L. culinaris*, *L. culinaris* subsp. *culinaris*, *L. culinaris* subsp. *orientalis*, *L. ervoides* and *L. nigricans*). Each sample is georeferenced and its DSr value is the mean of four years’ field trials. Each field trial followed a complete block design with 3 replications, artificially inoculating the samples and including frequent rows of susceptible checks to act as spreaders to ensure a high and uniform disease pressure. Disease Severity on mature plants in the field is assessed as a visual estimation of the leaf area covered by rust pustules, which is influenced by environmental factors. This value (DS) is standardized each year by expressing each DS value as a percentage of the highest one in each location that is set at 100% (DSr) (Sillero et al., 2017).

Each record of the filtered lentil CWR distribution database and of the calibration dataset was associated to the values of 65 bioclimatic, 35 edaphic and 18 geophysic variables at 2.5 arc-min resolution, corresponding to the sites of the populations. This information was obtained from the ecogeographic database of CAPFITOGEN3 (Parra-Quijano et al. 2020). Latitude and longitude were also added to the variable selection procedure. *SelecVar* function of CAPFITOGEN3 was used to estimate variable importance according to the random forest classification (RFC) and detected redundant variables through bivariate correlation analysis (Garcia et al., 2017). The first 15 variables of each bioclimatic, edaphic and geophysic component with the highest Mean Decrease Accuracy (MDA) values (Cutler et al., 2007; Rubio Teso et al., 2022) were checked for colinearity. Pairs of variables with Pearson correlation coefficient > |0.50| and p-value< 0.05 in the same ecogeographical component were identified and the variable with the lowest MDA removed. Hence, in the bioclimatic component, only annual mean temperature (°C) and annual precipitation (mm) were kept. In the edaphic component, four non-correlated variables were selected: bulk density (fine earth) of topsoil, topsoil available soil water capacity until wilting point, topsoil total exchangeable bases and topsoil sand fraction. Finally, in the geophysic component, three non-correlated variables were selected: annual solar radiation (*kJ*/*m* ^2^ *perday*), December solar radiation, and longitude. Further information and details about the generation and characteristics of this environmental database can be found in (Rubio Teso et al., 2022).

To identify the populations of lentil, crop wild relatives of Europe and Turkey that occur within protected areas, we considered the protected areas registered at the World Database of Protected Areas (WDPA) for this territory and those included in the Natura 2000 network. The file with the polygons of the WDPA was downloaded in April 2021 from the website ‘Explore the World’s Protected Areas’ (protectedplanet.net). The polygons of the Natura 2000 network were downloaded from the European Environment Agency website (https://www.eea.europa.eu/data-and-maps/data/natura-11/natura-2000-spatial-data/natura-2000-shapefile-1, last accessed 2021/07/20). The shapefile polygons from N2000 and WDPA obtained were merged into a single shapefile that contained all available protected areas in Europe and Turkey, using the function ‘join vector layers’ in QGIS v.3.18.2-Zürich (QGIS Org, 2021). All protected areas in the resulting shapefile were considered for the selection of candidate populations.

The dataset of lentil CWRs comprises 583 populations of four *Lens* taxa; 236 out of them grow inside a protected area (**Supplementary Table S1**).

## 3 Climate change models

Considering that climate change is expected to influence the evolutionary dynamics of CWR populations, one of the aims of this study was also to apply predictive characterization under future climate conditions. To do so, we incorporated climate change projections and Shared Socio-Economic Pathways (SSPs) to assess whether the environmental conditions that are likely to promote at present the presence of rust resistance in wild populations still remain in the forthcoming future. According to this, we combined the ecogeographic variables with the projected temperature and precipitation to quantitatively project the rust resistance of crop wild relative populations in future climate scenarios. A potential role of the results extracted here is their applicability in the selection of candidate populations (Stockwell and Peterson, 2002; Guisan and Thuiller, 2005; Araújo and Guisan, 2006).

The future climate Geographical Information System (GIS) layers were downloaded from the Worldclim database (http://www.worldclim.org/) at 2.5 arc-min resolution (around 5x5 km). From the available periods, we selected the 2021-2040 as the future climate scenario. We chose three global circulation models (GCMs) for climate change projections, produced by the Coupled Model Intercomparison Project Phase 6 (CMIP6) (O’Neill et al., 2016). For every GCM, we analysed three different shared socioeconomic pathways(SSPs), the most “pessimistic” or “conservative” scenario, a “balanced” scenario and an “optimistic” scenario, so we can cover the range of expectations to do a sensitivity analysis. Only the two bioclimatic variables that had been previously selected (Annual mean temperature and Annual mean precipitation) were considered.

## 4 Methods

### 4.1 Predictive characterization for evaluation accessions

In this study, we rely on the evaluated accessions that constituted the calibration dataset to carry out a quantitative predictive characterization using a machine learning regression approach. This dataset includes, on the one hand, the continuous range of the DSr as the dependent variable and the selected ecogeographical variables, at present time.

Rubio Teso et al. addressed the issue of predicting resistance to rust by means of the calibration method and a qualitative strategy with the same dataset (Rubio Teso et al., 2022). This method consisted of the binarization of DSr numerical values into qualitative values (resistant, susceptible), prior to prediction. According to this, accessions with the lowest DSr values were classified as rust-resistant, i.e., those located in the first decile of the distribution. The binarized levels of expression (0 = susceptible; 1 = resistant) were used as the dependent variable. Ecogeographical variables were the inputs to predict the binarized DSr resistance through nine classification algorithms. Then, the best predictor model was projected on the non-evaluated populations.

We followed a different path in this research, predicting the numerical value of DSr. We built and evaluated three different families of predictive models with the present time environmental values and DSr values for evaluated accessions: Ridge Regression (Hoerl, 1962, Random Forest (Breiman et al., 1984) and XGBoost (eXtreme Gradient Bosting) (Friedman, 2001; Chen and Guestrin, 2016).

#### 4.1.1 Regression models

DSr quantitative prediction is a regression problem with tabular data. We have implemented in Python the three regression models to tackle the task to identify populations potentially resistant to lentil rust.

Initially, we used the calibration dataset at present to train the models. From this dataset, we carry out a cleaning process in which we discard duplicate values and samples with incomplete variables, leaving us with a total of 255 samples. Considering the small sample size situation, in order to avoid cross-validation overfitting Ng et al. (1997) and achieving robust predictions for the different scenarios proposed, we address the following approach. We build 500 models that only differ in the random split of training and testing sets, including all variables. According to this, the dataset was split into different random train and test subsets, set to a 70/30 ratio. We train the model using the training subset and then we perform the predictions with the test subsets. Once the 500 models had been trained we collected all the predictions to better understand their distribution.

For visualization purposes we used the R programming language. A full list of the packages used is provided at the end of the methods section.

Ridge Regression is the simplest choice to achieve a balance between interpretability and precision. Since linear regression establishes a relationship between dependent variable and one or more independent variables that might be correlated, Ridge Regression imposes a penalty term on the size of the coefficients to overcome this issue, which is called multicollinearity (Gruber, 1998).

The aim of both Ordinary Least Squares (OLS) and Ridge Regression coefficients is to minimize the residual sum of squares (Saleh et al., 2019) and thus, the MSE. According to this, the penalty hyperparameter must be tuned so that model coefficients change in order to optimize the model error, by decreasing the residual sum of squares. As well as linear regression, the Ridge regressor explains the outcome as a function of multiple input variables. Thus, as a result, each input variable has an associated weight that will be positive or negative depending on its contribution to the model.

As well as linear regression, the Ridge regressor explains the outcome as a function of the multiple input variables. Thus, as a result, each input variable has an associated weight that will be positive or negative depending on its contribution to the model.

Random Forest Regression (RFR) is a tree-based ensemble method and belongs to the family of Classification and Regression Trees (CART). An ensemble method is a technique that combines the predictions from multiple machine learning algorithms together to make more accurate predictions than any individual model (Breiman, 2001).

RFR operates by constructing a multitude of decision trees, which are trained with a random subset of samples that have been drawn with replacement from the training sample. The number of variables included in each tree is limited to a percentage of the total variables that must be initially set. This ensures that the ensemble model does not rely too heavily on any individual variable, and makes fair use of all potentially predictive variables. As a result, the output estimation is the mean prediction of the individual trees.

Regarding interpretability, it is known that decision trees can be easily converted into rules which increase human interpretability of the results and explain why a decision was made. However, in the case of Random Forest it is not straightforward to find out the contribution of each of the variables (Rogers and Gunn, 2005). In Random Forests each Decision Tree is a set of internal nodes and leaves. In the internal node, the selected variable is used to make decision how to divide the data set into two separate sets with similar responses within. The variables for internal nodes are selected with some criterion, which for regression tasks is variance reduction. We can measure how each variable decrease the impurity of the split (the variable with highest decrease is selected for internal node). For each variable we can collect how on average it decreases the impurity. The average over all trees in the forest is the measure of the variable importance.

XGBoost (eXtreme Gradient Boosting) is another ensemble method that relies on the concept of gradient tree boosting. Boosting is a sequential algorithm that makes predictions for several rounds on the entire training sample and iteratively improves the performance of the algorithm with the information from the prior round’s prediction accuracy. However, XGBoost produces black box models, hard to visualize and tune compared to RFR (Agarwal and Das, 2020). Note that our aim is not to compare performance across a wide range of modelling techniques, but to show how different modelling approaches ranging from simple Ridge regression to more complex XGBoost can be explored within our framework.

#### 4.1.2 Variable engineering

To optimize the regressors, we followed a three-step process. In particular, we proceeded as follows:

- Variable selection. The environmental variables that might be the most relevant for explaining lentil taxa distribution were identified using a modified R script developed for the *SelecVar* tool from CAPFITOGEN3 (Parra-Quijano et al. 2020).
- Data normalization. Machine learning regressors typically require variables to have a close scale (Kotsiantis, 2011). The scale difference between variables can influence the performance of a ML regressor. Hence, we performed a data normalization scenario, namely standardization.
- Variable Importance. For the three regression models (Ridge Regression, Random Forests, and XGBoost), we ran a procedure after the training process to estimate variable importance. The importance of variables explaining rust resistance was carried out by analysing the weights assigned to the predictors for each model during the training step.

#### 4.1.3 Model evaluation

To assess the performance of regression models we computed the Root Mean Square Error (RMSE). Although Pearson correlation coefficient is widely used in quantitative genetics (González-Recio et al., 2014), Mean Squared Error (MSE) and RMSE yield better performance in model selection when the sample size is small and there is a high variance in the outcome variable (Oliveira et al., 2018; Waldmann, 2019). RMSE is a distance between the vectors of recorded values (*y_i_*) and predicted values $\left({\hat{y}}_{i}\right)$ (Chai and Draxler, 2014).


(1) $$RMSE=\sqrt{\frac{1}{N}\sum_{i=1}^{n}{\left(y_{i}-{\hat{y}}_{i}\right)}^{2}}$$

### 4.2 Non-evaluated projections under current conditions

As it was previously mentioned, regression models were initially trained using present time data for rust-resistance evaluated accessions. After that, we applied the best performing model to non-evaluated populations. This dataset had the same present-time ecogeographic variables than the calibration dataset.

We used the trained regression model to perform DSr projections on crop wild relative populations. Thus, we can identify those wild populations that are most likely to be resistant according to their predicted DSr value. Those populations for which DSr projection falls within the first quartile for the continuous range of rust-evaluated populations (*DSr* ≤ 30.48) and which are located in a protected area were selected as candidates to the long term *in situ* conservation.

### 4.3 DSr variation under climate change

Climate change models provide the future estimations for average temperature and yearly precipitation. According to this, we replaced both bioclimatic variables with future climate projections and applied these datasets as inputs to the predictive model. Those candidate populations to *in situ* conservation whose projected DSr under future climate conditions still falls within the first quartile of DSr distribution at present time were considered to be the populations most likely to retain the rust resistance trait, and, therefore, were selected as the most valuable populations for the establishment of genetic reserves.

List of statistical packages used

Python: python 3.8.8 (Van Rossum and Drake, 1995), matplotlib 3.3.4 (Hunter, 2007), numpy 1.20.1 (Harris et al., 2020), pandas 1.2.4 (Wes McKinney, 2010), seaborn 1.11.1 (Waskom, 2021), scikit-learn 0.24.1 (Pedregosa et al., 2011), verde 1.6.1 (Uieda, 2018), xgboost 1.4.2 (Chen and Guestrin, 2016). R: r-base 4.1.0 (R Core Team, 2020), countrycode 1.2.0 (Arel-Bundock et al., 2018), dplyr 3.4.0 (Wickham et al., 2022), forcats 0.5.1 (Wickham, 2021), gensysr 1.0.0 (Obreza, 2019), ggplot2 3.3.3 (Wickham, 2016), maps 3.3.0 (Brownrigg, 2018), readxl 1.3.1 (Wickham and Bryan, 2022), rgbif 0.9.8 (Chamberlain et al., 2017), scico 1.3.0 (Pedersen and Crameri, 2021), seewave 2.2.0 (Sueur et al., 2008).

## 5 Results

### 5.1 Predictive characterization

**Figure 1:**
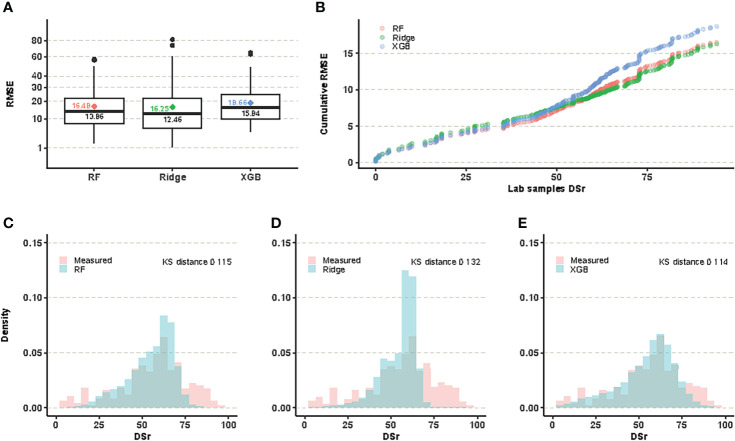
**(A)** RMSE of predicted DSr for evaluated samples for each of the three methods, average values are marked as color-filled diamonds. **(B)** The cumulative RMSE per sample is the sum of RMSE over the number of evaluated samples, and its value is equal to the average value for the last one. Random Forest is the best performer for low values of DSr. **(C–E)** plots compare the evaluated data distribution of DSr to the predicted one.

The training models generated with the three regression models rendered similar global results. **Figure 1A** shows the RMSE distributions for each of the three predictive models at present time, evaluated for the samples whose DSr values were known in advance. The Ridge model yielded the lowest median value, however this measure of centrality is not the only criterion to decide which is the most appropriate predictive model. Thus, the Ridge model showed a larger error spread than Random Forest, and, in addition, it was less accurate for small DSr values, precisely those corresponding to the most resistant samples and, therefore, the most valuable for conservation purposes (**Figure 1B**, **Supplementary Table S2**). Finally, a visual comparison of the distribution of the actual values and those predicted by the three methods reveals that the Ridge model tends to overpredict in the intermediate value range. **Figures 1C–E** include the values of the Kolmogorov-Smirnov distance which measures how the predicted and the evaluated values differ. Although the value of this difference is slightly lower for XGB than for Random Forest (0.114 vs. 0.116), the RMSE distribution of XGB showed a greater median value and error spread. For all these considerations, we selected Random Forest as the most suitable model to predict the DSr value of non-evaluated populations both at present and under different climate change scenarios.

We run the Variable Importance method for the Random Forest predictor, where results show that annual precipitation (mm) is the second most relevant, after Longitude. This fact becomes relevant specially when introducing the Climate Change models (**Table 1**), since annual precipitation was a variable susceptible to be modified, as well as annual average temperature that was at the fifth position in the variable importance ranking.

**Table 1:** Variable importance for the Random Forest predictor.

Variable	Importance
Longitude	0.2916
Annual precipitation	0.1623
Bulk density topsoil	0.0917
Available soil water capacity until wilting point	0.0866
Average annual temperature	0.0708
Topsoil sand fraction	0.0697
Topsoil total exchangeable bases	0.0651
Annual solar radiation	0.0615
Latitude	0.0548
Solar radiation December	0.0455

### 5.2 DSr variation under climate change

**Figure 2:**
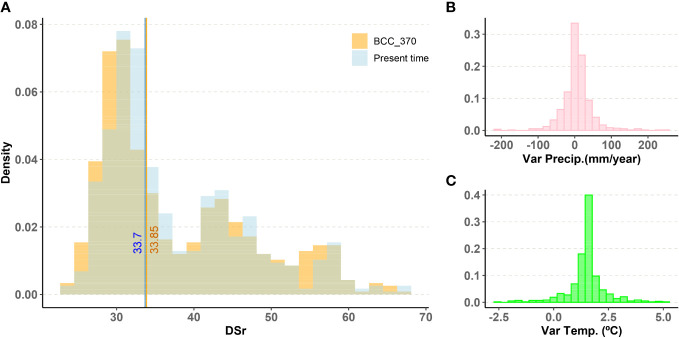
**(A)** Histogram of DSr prediction using Random Forest for non-evaluated wild populations in present time and in the future under BCC370 climate change model. Vertical lines mark the median value of each distribution. **(B)**, **(C)** Histograms of variation of average annual precipitation and average annual temperature between present time and future conditions under BCC370 climate model.

One of the main questions of this research is to assess how climate change may impact the ability of populations to maintain resistance to rust. An interesting result when comparing projections under current conditions and under climate change scenarios is that the global distribution of DSr values doesn’t drift in a clear direction. **Figure 2A** shows that the distribution of the DSr value is very similar for the present and future predictions under the BCC370 model. The median remains almost unchanged, going from 33.70 to 33.85.

We have built the Random Forest predictor for all the climate change models described in methods. In what follows we always refer to the Random Forest regression under the conditions of change of the BCC370 model.

The variation in annual precipitation is weakly positive, with a median increase of 6 mm (**Figure 2B**). This change is highly concentrated around the median with -9 mm variation at the limit of the first quartile and 22 mm at the limit of the third quartile. In other words, locations with the lowest precipitation tended to lower their precipitation even further, whereas the other locations increased their precipitation, especially those which initially had the highest precipitation. Average annual temperature increases, on the other hand, are remarkable with values 1.28 °C, 1.56 °C and 1.71 °C for the first quartile, the median and the third quartile (**Figure 2C**).

**Figure 3:**
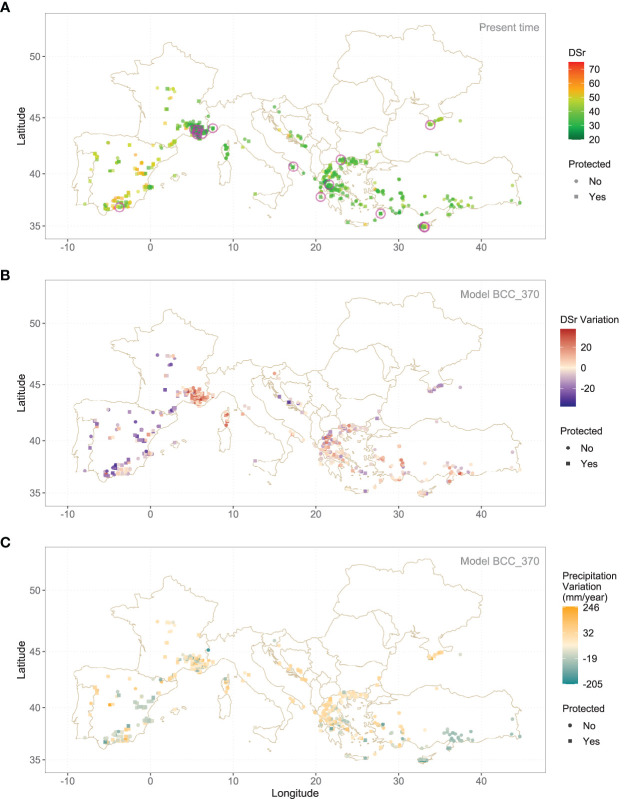
**(A)** DSr predicted median value, using Random Forest, for wild accessions under present climate conditions. **(B)** Variation of the same parameter according to BCC370 model. Encircled samples in **(A)** show a low DSr (first quartile) both in present time and in the future and are located inside a protected area. **(C)** Variation of annual precipitation.

The highest values of sensitivity to rust are found in some locations in the interior of the Iberian Peninsula, in regions with very hot and dry summers where rust cannot thrive (**Figure 3A**). Something similar occurs in the interior of France, but in this same country there is a cluster of potentially resistant populations in the final course of the Rhone, an area with much higher humidity conditions. Proximity to sea seems to foster populations with low DSr, as it happens in Greece, the Anatolian shoreline and Southern Crimea. **Figure 3B** shows the map of DSr variation with respect to the present time for the same wild accessions using Model BCC_370 instead. Spatial patterns are easy to spot. According to this, DSr values will decrease in the mountains of the Iberian Peninsula, but will be higher in the Rhone region and Southern Greece (See **Supplementary Figures S2** and **S3** for details). Annual precipitation is the main driver of these changes, with a general trend to drought. **Figure 3C** shows the variation of precipitation according to the BCC_370 model.

**Supplementary Table S1** shows the number of populations whose present DSr value falls under the first quartile for the present time (*DSr* ≤ 30.48) and are inside a protected area: 14 belong to *Lens ervoides* and 36 to *Lens nigricans*. Out of these 51 populations there are 16 that have a DSr value under 30.48 under the BCC370 hypothesis of Climate Change, 7 of *Lens ervoides* and 9 of *Lens nigricans*. They are encircled in magenta in **Figure 3B**. They are the most valuable populations for *in situ* conservation and are listed in **Table 2**.

**Table 2:** Selected subset of wild lentil populations for *in situ* conservation, occurring in protected areas and most likely to be rust-resistant at present and in the future according to the tested climate change projection models.

DSr	DSr	Long.	Lati.	Species	Site
Present	Future				
29.57	28.84	33.16	34.88	*Lens ervoides*	Nicosia District, Ora, Greece
29.22	30.35	33.09	34.91	*Lens ervoides*	Nicosia District, Palaichori, Greece
28.96	29.89	27.87	36.20	*Lens ervoides*	Rodos, Mt. Attaviros, Greece PN de las Sierras de Tejeda, Almijara y Alhama, Spain
29.28	26.95	-3.72	36.82	*Lens ervoides*	
27.80	28.78	20.64	37.82	*Lens ervoides*	Zakynthos, Porto Vromi, Greece
29.07	24.21	21.66	38.98	*Lens ervoides*	Evrytania, NW Nea Viniani, Greece
29.24	29.37	33.83	44.40	*Lens ervoides*	Near Black Sea and Sanatome, Crimea
27.95	29.16	17.25	40.67	*Lens nigricans*	Puglia, 14 km from Massafra, Italy
30.04	24.68	23.02	41.29	*Lens nigricans*	Serrai, S Kato Poroia, Greece
26.49	29.37	5.66	43.54	*Lens nigricans*	Brians, France
28.55	28.95	6.28	43.61	*Lens nigricans*	Ampus, France
28.84	28.08	5.67	43.72	*Lens nigricans*	Mirabeau, France
28.52	26.61	5.68	43.90	*Lens nigricans*	Reillane, France
30.28	27.10	5.81	43.99	*Lens nigricans*	Forcalquier, France
27.54	30.11	5.31	44.00	*Lens nigricans*	Lioux, France
30.22	26.94	7.56	44.11	*Lens nigricans*	Tende, France

## 6 Discussion

In this study, we addressed the pressing problem of the lack of adaptive resilience in modern cultivars in the context of climate change and the need to identify crop wild relative populations that might provide the genetic diversity needed to obtain specific traits. Rust is a severe disease for lentil production and breeding with resistant CWRs is a convenient way to reduce this problem but systematic straight identification of wild samples that have evolved to resist the fungus is not possible because of the expensive and time-consuming method to estimate Disease Severity. We tackled this problem by building a Machine Learning model using a rich dataset of calibrated samples grown under controlled environmental conditions. We used the model to predict the sensitivity to rust of a set of samples of wild relatives of *Lens culinaris* subsp. *culinaris*, in the present time and under 9 scenarios of Climate Change. As DSr is a quantitave measurement, we have gone straight to a regressive model instead of a qualitative binarized approach as in previous works. Our goal was to identify natural CWR populations in protected areas, that are likely to be resistant to rust at present time and to maintain this trait in the future due to the maintenance of selective pressures (i.e., have a projected low DSr at present time and in the future scenarios of climate change models). A quantitative estimation is better suited for the purpose of finding extremely resistant accessions.

With this methodological approach we aimed to answer three questions. The first one is which variables have more impact the model. Variable importance analysis revealed that Longitude is the most relevant one. This fact comes as no surprise given the East-West distribution of lentil wild species populations across the Mediterranean basin (Ladizinsky et al., 1983; Ladizinsky et al., 1984), and the evidences of westward migration from Near East of other wild *Fabaceae* like wild peas (Smỳkal et al., 2017; Hellwig et al., 2022) or wild lupins (Mousavi-Derazmahalleh et al., 2018). Rust sensitivity is higher in the West Mediterranean basin, with extreme values in inner areas of the Iberian Peninsula and France, where the conditions are less prone to rust development. These results are in line with (Singh et al., 2014) who experimentally evaluated 405 wild lentil accessions and identified 27 promising rust-resistant populations which were mostly located in the Eastern Mediterranean (Syria and Turkey). Besides longitude, annual precipitation, bulk density topsoil, available soil water capacity and annual average temperature are the most relevant for the RFR predictor.

In response to the second question we identify 51 populations whose DSr values are under the first quartile in present time and grow inside a protected area. These are good rust-resistant candidate populations which should be evaluated for this trait and could be easily conserved *in situ*.

The third question constituting a relevant landmark of this work was to identify which of those 51 populations are likely to maintain the environmental conditions that are favourable for the occurrence of rust-resistant genotypes under the projected Climate Change scenarios. Although longitude is the variable with the greatest weight in the prediction of our model, this variable is constant through time and not affected by climate change. Annual precipitation and average annual temperature were the variables that had an impact in the different scenarios on the future sensitivity to rust. The effect on the variation of DSr was similar for them all (**Supplementary Figure S1**; **Supplementary Table S3**), possibly because of the short span over which the change is projected (year 2040). Locations with the lowest precipitation tended to lower their precipitation even further, whereas the other locations increased their precipitation, especially those which initially had the highest precipitation. Average annual temperature increases, on the other hand, are remarkable with values 1.28 °C, 1.56 °C and 1.71 °C for the first quartile, the median and the third quartile. This could suggest that rust resistance is not affected by the changes in annual precipitation and mean temperature, but quite the opposite, we found clear patterns of DSr variation by geographic area. Annual precipitation was found to be the main driver of change and those regions with increased precipitation were associated to conditions more favourable for rust-resistant populations (lower DSr values). In particular, the accessions of South Crimea, the Iberian plateaus and Western France would be the ones that experiment a greater increase in precipitation and consequently a decrease in projected DSr. Just the opposite would happen on the Southern shore of Anatolia, where precipitation is going to decrease sharply reducing the chances of finding rust resistant genotypes in those locations.

We identified 16 populations in protected areas with DSr values below 30.48 (first quartile of the distribution of the DSr projection) at the present time and under the future environmental conditions (**Table 2**). Eight populations are located in the Eastern Mediterranean basin (6 belonging to *Lens ervoides* and 2 to *Lens nigricans*). In the Western basin, 7 populations are in the South of France (all of them belonging to *Lens nigricans*) and only 1 is in the Iberian Peninsula (*Lens ervoides*). There is a remarkable difference among the CWR species. While 7 out of 14 populations of *Lens ervoides* selected at present time will remain of high interest in the future from the rust-resistance point of view, only 9 of 37 populations of *Lens nigricans* fall within this category. They all share a common geographic feature, they are very close to the coast, and 5 of them are on islands (Rhodes, Zakhyntos, Crete and Cyprus). There are not populations of *Lens lamottei* and *Lens culinaris* subsp. *orientalis* in the selected subset of high interest populations for *in situ* conservation, but the initial number of populations for these species was small compared to the other species. This fact suggests an interest for focusing future field work on these underrepresented taxa.

## 7 Conclusion

Crop Wild Relatives of *Lens culinaris* subsp. *culinaris* are a source of genetic resistance to a commonrust disease caused by the fungus *Uromyces viciae-fabae*. The quantitative field evaluation of rust resistance is a hard, expensive, and time-consuming task. The use of Machine Learning approaches provided a way to mitigate this obstacle, using a carefully built calibration set. Our results identified 16 populations that are likely to be resistant to rust, occur in protected areas, and are expected to be resilient under predicted Climate Change conditions. Thus, they are sound candidates for the establishment of genetic reserves for *in situ* conservation. Further characterization by field evaluation of these populations is needed to check the validity of Machine Learning predictions and improve the genetic value of the calibration set. The same method may be extended to predict pest and pathogen resistance traits of CWRs of other crops.

## Data availability statement

Publicly available datasets were analyzed in this study. This data can be found here: https://zenodo.org/record/6883274.

## Author contributions

Conceptualization: IC-G, MLRT, JI, JG, JG-A. Data curation: DR, MLRT, JI. Funding acquisition: JI, JG, DR. Methodology: IC-G, JG-A, JG, JI. Machine learning models: IC-G, JG-A, MLRT. Visualization: JG-A. Writing, original draft: IC-G, JG-A, JI, JG. Writing, review & editing: IC-G, MLRT, JG-A, JG, DR, JI. All authors contributed to the article and approved the submitted version.

## Funding

This research was partially funded by the Farmer’s Pride project: Networking, Partnerships, and Tools to Enhance *in situ* Conservation of European Plant Genetic Resources, a 3-year EU-funded project funded by the Horizon 2020 Framework Programme of the European Union, grant agreement no. 774271. J.G. acknowledges financial support provided by the Ministerio de Ciencia, Innovación y Universidades (*PGC*2018-093854-*B*-100). This research was partially funded too by *I* + *D* + *i* Plan Andaluz Investigación LEGAND project (P20_0986).

## Conflict of interest

The authors declare that the research was conducted in the absence of any commercial or financial relationships that could be construed as a potential conflict of interest.

## Publisher’s note

All claims expressed in this article are solely those of the authors and do not necessarily represent those of their affiliated organizations, or those of the publisher, the editors and the reviewers. Any product that may be evaluated in this article, or claim that may be made by its manufacturer, is not guaranteed or endorsed by the publisher.

## References

[B1] Adhikari U. Nejadhashemi A. P. Woznicki S. A. (2015). Climate change and Eastern Africa: A review of impact on major crops. Food Energy Secur. 4, 110–132. doi: 10.1002/fes3.61

[B2] Agarwal N. Das S. (2020). “Interpretable Machine Learning Tools: A Survey. In Proceedings of the IEEE Symposium Series on Computational Intelligence (SSCI) Canberra, Australia, 1–4, 1528–1534

[B3] Amouzoune M. Amri A. Benkirane R. Kehel Z. Al-Jaboobi M. Moulakat A.. (2022). Mining and predictive characterization of resistance to leaf rust (*Puccinia hordei otth*) using two subsets of barley genetic resources. Genet. Resour. Crop Evol. 69, 839–853. doi: 10.1007/s10722-021-01268-4

[B4] Anderson R. Bayer P. E. Edwards D. (2020). Climate change and the need for agricultural adaptation. Curr. Opin. Plant Biol. 56, 197–202. doi: 10.1016/j.pbi.2019.12.006 32057694

[B5] Araújo M. Guisan A. (2006). Araujo mb, guisan a. five (or so) challenges for species distribution modelling. journal of biogeography. J. Biogeogr. 33, 1677–1688. doi: 10.1111/j.1365-2699.2006.01584.x

[B6] Arel-Bundock V. Enevoldsen N. Yetman C. (2018). Countrycode: An r package to convert country names and country codes. J. Open Source Software. 3, 848. doi: 10.21105/joss.00848

[B7] Arojju S. K. Conaghan P. Barth S. Milbourne D. Casler M. D. Hodkinson T. R.. (2018). Genomic prediction of crown rust resistance in lolium perenne. BMC Genet. 19, 1–10. doi: 10.1186/s12863-018-0613-z 29843601 PMC5975627

[B8] Bari A. Amri A. Street K. Mackay M. De Pauw E. Sanders R.. (2014). Predicting resistance to stripe (yellow) rust (*Puccinia striiformis*) in wheat genetic resources using focused identification of germplasm strategy. J. Agric. Sci. 152, 906–916. doi: 10.1017/S0021859613000543

[B10] Barilli E. Sillero J. C. Fernández-Aparicio M. Rubiales D. (2009). Identification of resistance to *Uromyces pisi* (pers.) wint. in *Pisum* spp. germplasm. Field Crops Res. 114, 198–203. doi: 10.1016/j.fcr.2009.07.017

[B9] Bari A. Street K. Mackay M. Endresen D. T. F. De Pauw E. Amri A. (2012). Focused identification of germplasm strategy (figs) detects wheat stem rust resistance linked to environmental variables. Genet. Resour. Crop Evol. 59, 1465–1481. doi: 10.1007/s10722-011-9775-5

[B11] Breiman L. (2001). Random forests. Mach. Learn. 45, 5–32. doi: 10.1023/A:1010933404324

[B12] Breiman L. Friedman J. Stone C. J. Olshen R. A. (1984). Classification and Regression Trees. (1st ed.). New York: Routledge. doi: 10.1201/9781315139470

[B13] Brownrigg R. (2018) Package ‘maps’. Available at: https://cran.r-project.org/package=maps.

[B14] Castañeda-Álvarez N. P. Khoury C. K. Achicanoy H. A. Bernau V. Dempewolf H. Eastwood R. J.. (2016). Global conservation priorities for crop wild relatives. Nat. Plants 2, 1–6. doi: 10.1038/nplants.2016.22 27249561

[B15] Chai T. Draxler R. R. (2014). Root mean square error (rmse) or mean absolute error (mae). Geosci. Model. Dev. Discussions. 7, 1525–1534. doi: 10.5194/gmdd-7-1525-2014

[B16] Chamberlain S. Ram K. Barve V. Mcglinn D. Chamberlain M. S. (2017) Package ‘rgbif’. Available at: https://cran.r-project.org/package=rgbif.

[B17] Chen T. Guestrin C. (2016). “Xgboost: A scalable tree boosting system,” in Proceedings of the 22nd acm sigkdd international conference on knowledge discovery and data mining. (ACM), 785–794.

[B18] Cramer W. Guiot J. Fader M. Garrabou J. Gattuso J.-P. Iglesias A.. (2018). Climate change and interconnected risks to sustainable development in the mediterranean. Nat. Climate Change 8, 972–980. doi: 10.1038/s41558-018-0299-2

[B19] Cutler D. R. Edwards T. C. Jr. Beard K. H. Cutler A. Hess K. T. Gibson J.. (2007). Random forests for classification in ecology. Ecology 88, 2783–2792. doi: 10.1890/07-0539.1 18051647

[B20] Dempewolf H. Baute G. Anderson J. Kilian B. Smith C. Guarino L. (2017). Past and future use of wild relatives in crop breeding. Crop Sci. 57, 1070–1082. doi: 10.2135/cropsci2016.10.0885

[B21] Dufresne J.-L. Foujols M.-A. Denvil S. Caubel A. Marti O. Aumont O.. (2013). Climate change projections using the ipsl-cm5 earth system model: from cmip3 to cmip5. Climate Dynamics. 40, 2123–2165. doi: 10.1007/s00382-012-1636-1

[B22] El Haddad N. Kabbaj H. Zaïm M. El Hassouni K. Tidiane Sall A. Azouz M.. (2021). Crop wild relatives in durum wheat breeding: Drift or thrift? Crop Sci. 61, 37–54. doi: 10.1002/csc2.20223

[B23] Endresen D. T. F. (2010). Predictive association between trait data and ecogeographic data for nordic barley landraces. Crop Sci. 50, 2418–2430. doi: 10.2135/cropsci2010.03.0174

[B24] Endresen D. T. F. Street K. Mackay M. Bari A. Amri A. De Pauw E.. (2012). Sources of resistance to stem rust (ug99) in bread wheat and durum wheat identified using focused identification of germplasm strategy. Crop Sci. 52, 764–773. doi: 10.2135/cropsci2011.08.0427

[B25] Fellmann T. Witzke P. Weiss F. Van Doorslaer B. Drabik D. Huck I.. (2018). Major challenges of integrating agriculture into climate change mitigation policy frameworks. Mitigation. Adapt. Strat. Global Change 23, 451–468. doi: 10.1007/s11027-017-9743-2 PMC6054014 30093833

[B26] Food and of the United Nations A. O (2014). Herramientas CAPFITOGEN: Programa para El fortalecimiento de las capacidades en programas nacionales de recursos (FAO).

[B27] Friedman J. H. (2001). Greedy function approximation: a gradient boosting machine. Ann. Stat, 29, 1189–1232. doi: 10.1214/aos/1013203451

[B28] Fu Y.-B. (2017). The vulnerability of plant genetic resources conserved ex situ. Crop Sci. 57, 2314–2328. doi: 10.2135/cropsci2017.01.0014

[B29] Garcia R. M. Parra-Quijano M. Iriondo J. M. (2017). Identification of ecogeographical gaps in the spanish aegilops collections with potential tolerance to drought and salinity. PeerJ 5, e3494. doi: 10.7717/peerj.3494 28761779 PMC5534164

[B30] González-Recio O. Rosa G. J. Gianola D. (2014). Machine learning methods and predictive ability metrics for genome-wide prediction of complex traits. Livestock. Sci. 166, 217–231. doi: 10.1016/j.livsci.2014.05.036

[B31] Gruber M. (1998). Improving efficiency by shrinkage: The James-stein and ridge regression estimators Vol. 156 (New York, CRC Press).

[B32] Guisan A. Thuiller W. (2005). Predicting species distribution: Offering more than simple habitat models. Ecol. Lett. 8, 993–1009. doi: 10.1111/j.1461-0248.2005.00792.x 34517687

[B33] Hannah L. (2010). A global conservation system for climate-change adaptation. Conserv. Biol. 24, 70–77. doi: 10.1111/j.1523-1739.2009.01405.x 20121843

[B34] Harris C. R. Millman K. J. van der Walt S. J. Gommers R. Virtanen P. Cournapeau D.. (2020). Array programming with NumPy. Nature 585, 357–362. doi: 10.1038/s41586-020-2649-2 32939066 PMC7759461

[B35] Hellwig T. Abbo S. Ophir R. (2022). Drivers of genetic differentiation and recent evolutionary history of an eurasian wild pea. J. Biogeogr. 49, 794–808. doi: 10.1111/jbi.14274

[B36] Heywood V. Casas A. Ford-Lloyd B. Kell S. Maxted N. (2007). Conservation and sustainable use of crop wild relatives. Agricult. Ecosyst. Environ. 121, 245–255. doi: 10.1016/j.agee.2006.12.014

[B37] Hoerl A. (1962). Application of ridge analysis to regression problems. Chem. Eng. Prog. 58, 54–59.

[B38] Howden S. M. Soussana J.-F. Tubiello F. N. Chhetri N. Dunlop M. Meinke H. (2007). Adapting agriculture to climate change. Proc. Natl. Acad. Sci. 104, 19691–19696. doi: 10.1073/pnas.0701890104 18077402 PMC2148359

[B41] Hunter J. D. (2007). Matplotlib: A 2d graphics environment. Computing. Sci. Eng. 9, 90–95. doi: 10.1109/MCSE.2007.55

[B39] Hunter D. (2012). Crop wild relatives: a manual of in situ conservation (Routledge, London), 452.

[B40] Hunter D. Maxted N. Heywood V. Kell S. Borelli T. (2012). Protected areas and the challenge of conserving crop wild relatives. Parks 18, 87.

[B42] Iriondo J. M. Maxted N. Dulloo M. E. (2008). Conserving plant genetic diversity in protected areas: population management of crop wild relatives (Wallingford (UK), CAB International).

[B43] Khoury C. K. Brush S. Costich D. E. Curry H. A. de Haan S. Engels J. M.. (2022). Crop genetic erosion: Understanding and responding to loss of crop diversity. New Phytol. 233, 84–118. doi: 10.1111/nph.17733 34515358

[B44] Khoury C. K. Carver D. Greene S. L. Williams K. A. Achicanoy H. A. Schori M.. (2020). Crop wild relatives of the united states require urgent conservation action. Proc. Natl. Acad. Sci. 117, 33351–33357. doi: 10.1073/pnas.2007029117 33318205 PMC7776777

[B45] Kotsiantis S. (2011). Feature selection for machine learning classification problems: a recent overview. Artif. Intell. Rev. 42, 157–176. doi: 10.1007/s10462-011-9230-1

[B46] Labokas J. Maxted N. Kell S. Brehm J. M. Iriondo J. M. (2018). Development of national crop wild relative conservation strategies in european countries. Genet. Resour. Crop Evol. 65, 1385–1403. doi: 10.1007/s10722-018-0621-x

[B47] Ladizinsky G. Braun D. Goshen D. Muehlbauer F. (1984). The biological species of the genus. Lens. L. Bot. Gazette. 145, 253–261. doi: 10.1086/337454

[B48] Ladizinsky G. Braun D. Muehlbauer F. (1983). Evidence for domestication of *Lens nigricans* (M. bieb.) godron in s Europe. Bot. J. Linn. Soc. 87, 169–176. doi: 10.1111/j.1095-8339.1983.tb00988.x

[B49] Liber M. Duarte I. Maia A. T. Oliveira H. R. (2021). The history of lentil *(Lens culinaris subsp. culinaris)* domestication and spread as revealed by genotyping-by-sequencing of wild and landrace accessions. Front. Plant Sci. 12, 628439. doi: 10.3389/fpls.2021.628439 33841458 PMC8030269

[B500] Maxted N. Iriondo J. M. Dulloo M. E. Lane A. (2008). The integration of PGR Conservation with Protected Area Management. In: Conserving plant genetic diversity in protected areas. Population management of crop wild relatives Iriondo J. M. Dulloo M. E. Maxted N. (Wallingford. (UK): CAB International) 1–22.

[B501] Maxted N. Ford-Lloyd B.V. Kell S.P. Iriondo J.M. Dulloo Turok M.E. J. (2008). Population management of crop wild relatives (Wallingford. (UK): CAB International) 1–22.

[B50] Maxted N. Ford-Lloyd B.V. Kell S.P. Iriondo J.M. Dulloo Turok M.E. J. (eds.) (2008). P Crop Wild Relative Conservation and Use (Wallingford. (UK): CAB International). 10.1186/1471-2105-9-116 PMC2375123 18298814

[B51] Maxted N. Iriondo J. M. Dulloo M. E. Lane A. (2008). Introduction: The integration of PGR Conservation with Protected Area Management. In: Conserving plant genetic diversity in protected areas. Population management of crop wild relatives Iriondo J. M. Dulloo M. E. Maxted N. (Wallingford. (UK): CAB International) pp. 1–22.

[B52] Maxted N. Kell S. (2009). Establishment of a global network for the situ conservation of crop wild relatives: Status and needs (Rome, Italy: FAO Commission on Genetic Resources for Food and Agriculture).

[B53] Maxted N. Kell S. Ford-Lloyd B. Dulloo E. Toledo Á. (2012). Toward the systematic conservation of global crop wild relative diversity. Crop Sci. 52, 774–785. doi: 10.2135/cropsci2011.08.0415

[B55] Meilleur B. A. Hodgkin T. (2004). *In situ* conservation of crop wild relatives: status and trends. Biodivers. Conserv. 13, 663–684. doi: 10.1023/B:BIOC.0000011719.03230.17

[B56] Mousavi-Derazmahalleh M. Bayer P. E. Nevado B. Hurgobin B. Filatov D. Kilian A.. (2018). Exploring the genetic and adaptive diversity of a pan-mediterranean crop wild relative: narrow-leafed lupin. Theor. Appl. Genet. 131, 887–901. doi: 10.1007/s00122-017-3045-7 29353413 PMC5852200

[B57] Negussie T. Pretorius Z. (2012). Lentil rust: Present status and future prospects. Crop Prot. 32, 119–128. doi: 10.1016/j.cropro.2011.11.004

[B58] Ng A. Y. (1997). “Preventing” overfitting” of cross-validation data,” in Machine Learning: Proceedings of the Fourteenth International Conference (ICML ’97), vol. 97, 245–253.

[B59] Obreza M. (2019) Preventing “overfitting” of cross-validation data. Machine Learning: Proceedings of the Fourteenth International Conference (ICML ’97), 245–253

[B60] Olesen J. E. Bindi M. (2002). Consequences of climate change for european agricultural productivity, land use and policy. Eur. J. Agron. 16, 239–262. doi: 10.1016/S1161-0301(02)00004-7

[B61] Oliveira M. Pereira G. Ferreira E. Santos J. Knezevic S. Werle R. (2018). Additive design: the concept and data analysis. Weed. Res. 58, 338–347. doi: 10.1111/wre.12317

[B64] O’Neill B. C. Kriegler E. Riahi K. Ebi K. L. Hallegatte S. Carter T. R.. (2014). A new scenario framework for climate change research: the concept of shared socioeconomic pathways. Clim. Change 122, 387–400. doi: 10.1007/s10584-013-0905-2

[B62] O’Neill B. C. Tebaldi C. Van Vuuren D. P. Eyring V. Friedlingstein P. Hurtt G.. (2016). The scenario model intercomparison project (scenariomip) for cmip6. Geosci. Model. Dev. 9, 3461–3482. doi: 10.5194/gmd-9-3461-2016

[B63] QGIS Org (2021). Qgis geographic information system (QGIS Association). Open source geospatial foundation project. Available at: https://qgis.org/es.

[B502] Parra-Quijano M. Iriondo J. M. Torres M. E. López F. Maxted N. Kell S. (2020). CAPFITOGEN3. A Toolbox for the Conservation and Promotion of the use of Agricultural Biodiversity. (Bogotá, Colombia: Universidad Nacional de Colombia. Bogotá campus – Faculty of Agricultural Sciences). Available online at: http://www.capfitogen.net/es/acceso/manuales/

[B65] Pedersen T. L. Crameri F. (2021) Package ‘scico’. Available at: https://cran.r-project.org/package=scico.

[B66] Pedregosa F. Varoquaux G. Gramfort A. Michel V. Thirion B. Grisel O.. (2011). Scikit-learn: Machine learning in python. J. Mach. Learn. Res. 12, 2825–2830. Available at: https://www.jmlr.org/papers/volume12/pedregosa11a/pedregosa11a.pdf

[B68] Rauf S. da Silva J. T. Khan A. A. Naveed A. (2010). Consequences of plant breeding on genetic diversity. Int. J. Plant Breed. 4, 1–21.

[B69] Ray D. K. West P. C. Clark M. Gerber J. S. Prishchepov A. V. Chatterjee S. (2019). Climate change has likely already affected global food production. PloS One 14, e0217148. doi: 10.1371/journal.pone.0217148 31150427 PMC6544233

[B67] R Core Team (2020). R: A language and environment for statistical computing (Vienna, Austria: R Foundation for Statistical Computing). Available at: https://www.R-project.org/.

[B70] Ren J. Li Z. Wu P. Zhang A. Liu Y. Hu G.. (2021). Genetic dissection of quantitative resistance to common rust (*Puccinia sorghi*) in tropical maize (*zea mays* l.) by combined genome-wide association study, linkage mapping, and genomic prediction. Front. Plant Sci. 12, 1338. doi: 10.3389/fpls.2021.692205 PMC8284423 34276741

[B71] Rogers J. Gunn S. (2005). “Identifying feature relevance using a random forest,” in International statistical and optimization perspectives workshop” subspace, latent structure and feature selection (Berlin, Springer), 173–184.

[B72] Rubiales D. Castillejo M. Madrid E. Barilli E. Rispail N. (2011). Legume breeding for rust resistance: lessons to learn from the model *Medicago truncatula* . Euphytica 180, 89–98. doi: 10.1007/s10681-011-0367-4

[B73] Rubiales D. Fondevilla S. Fernández-Aparicio M. (2020). Development of pea breeding lines with resistance to *Orobanche crenata* derived from pea landraces and wild *Pisum* spp. Agronomy 11, 36. doi: 10.3390/agronomy11010036

[B74] Rubiales D. Rojas-Molina M. M. Sillero J. C. (2013). Identification of pre-and posthaustorial resistance to rust (*Uromyces viciae-fabae*) in lentil (*Lens culinaris*) germplasm. Plant Breed. 132, 676–680. doi: 10.1111/pbr.12096

[B75] Rubio Teso M. L. Álvarez Muñiz C. Gaisberger H. Kell S. Lara-Romero C. Magos Brehm J.. (2020). In situ plant genetic resources in Europe: crop wild relatives. Farmer’s Pride Project, https://more.bham.ac.uk/farmerspride/wp-content/uploads/sites/19/2020/10/D1.2_In_situ_PGR_in_Europe_crop_wild_relatives.pdf

[B76] Rubio Teso M. L. Lara-Romero C. Rubiales D. Parra-Quijano M. Iriondo J. M. (2022). Searching for abiotic tolerant and biotic stress resistant wild lentils for introgression breeding through predictive characterization. Front. Plant Sci. 13. doi: 10.3389/fpls.2022.817849 PMC8928559 35310661

[B77] Rufo R. Alvaro F. Royo C. Soriano J. M. (2019). From landraces to improved cultivars: Assessment of genetic diversity and population structure of mediterranean wheat using snp markers. PloS One 14, e0219867. doi: 10.1371/journal.pone.0219867 31306459 PMC6629082

[B78] Saleh A. M. E. Arashi M. Kibria B. G. (2019). Theory of ridge regression estimation with applications. 285 (Oxford (UK): John Wiley & Sons).

[B79] Sánchez R. M. G. Parra-Quijano M. Greene S. Iriondo J. M. (2019). Predictive characterisation identifies global sources of acyanogenic germplasm of a key forage species. Crop Pasture Sci. 70, 546–554. doi: 10.1071/CP18346

[B80] Seiler G. J. Qi L. L. Marek L. F. (2017). Utilization of sunflower crop wild relatives for cultivated sunflower improvement. Crop Sci. 57, 1083–1101. doi: 10.2135/cropsci2016.10.0856

[B81] Sillero J. C. Rojas-Molina M. M. Emeran A. A. Kharrat M. Winkler J. Khan H. R.. (2017). Identification and multi-environment validation of resistance to rust (*Uromyces viciae-fabae*) in *vicia faba* . Crop Pasture Sci. 68, 1013–1023. doi: 10.1071/CP17099

[B82] Singh M. Bisht I. S. Kumar S. Dutta M. Bansal K. C. Karale M.. (2014). Global wild annual lens collection: a potential resource for lentil genetic base broadening and yield enhancement. PloS One 9, e107781. doi: 10.1371/journal.pone.0107781 25254552 PMC4177869

[B54] Smith P. Howden M. Krug M-D Mbow C. Pörtner H.O. Reisinger A. (2017). Special report on climate change, desertification, land degradation, sustainable land management, food security, and greenhouse gas fluxes in terrestrial ecosystems (sr2). Geneva: IPCC, 650

[B83] Smỳkal P. Hradilová I. Trněnỳ O. Brus J. Rathore A. Bariotakis M.. (2017). Genomic diversity and macroecology of the crop wild relatives of domesticated pea. Sci. Rep. 7, 1–12. doi: 10.1038/s41598-017-17623-4 29234080 PMC5727218

[B84] Stockwell D. R. Peterson A. (2002). Effects of sample size on accuracy of species distribution models. Ecol. Model. 148, 1–13. doi: 10.1016/S0304-3800(01)00388-X

[B85] Sueur J. Aubin T. Simonis C. (2008). Seewave: a free modular tool for sound analysis and synthesis. Bioacoustics 18, 213–226. doi: 10.1080/09524622.2008.9753600

[B86] Triviño M. Kujala H. Araújo M. B. Cabeza M. (2018). Planning for the future: identifying conservation priority areas for iberian birds under climate change. Landscape Ecol. 33, 659–673. doi: 10.1007/s10980-018-0626-z

[B87] Uieda L. (2018). Verde: Processing and gridding spatial data using green’s functions. J. Open Source Software. 3, 957. doi: 10.21105/joss.00957

[B88] Van de Wouw M. van Hintum T. Kik C. van Treuren R. Visser B. (2010). Genetic diversity trends in twentieth century crop cultivars: a meta analysis. Theor. Appl. Genet. 120, 1241–1252. doi: 10.1007/s00122-009-1252-6 20054521 PMC2839474

[B89] Van Rossum G. Drake F. L. Jr (1995). Python Reference manual (Amsterdam, Centrum voor Wiskunde en Informatica Amsterdam).

[B90] van Treuren R. Hoekstra R. van Hintum T. J. (2017). Inventory and prioritization for the conservation of crop wild relatives in the netherlands under climate change. Biol. Conserv. 216, 123–139. doi: 10.1016/j.biocon.2017.10.003

[B91] Vincent H. Amri A. Castañeda-Álvarez N. P. Dempewolf H. Dulloo E. Guarino L.. (2019). Modeling of crop wild relative species identifies areas globally for *in situ* conservation. Commun. Biol. 2, 1–8. doi: 10.1038/s42003-019-0372-z 31044161 PMC6478866

[B92] Voldoire A. Sanchez-Gomez E. Salas y Mélia D. Decharme B. Cassou C. Sénési S.. (2013). The cnrm-cm5. 1 global climate model: description and basic evaluation. Climate Dynamics. 40, 2091–2121. doi: 10.1007/s00382-011-1259-y

[B93] Waldmann P. (2019). On the use of the pearson correlation coefficient for model evaluation in genome-wide prediction. Front. Genet. 10. doi: 10.3389/fgene.2019.00899 PMC6781837 31632436

[B94] Waskom M. L. (2021). Seaborn: statistical data visualization. J. Open Source Software. 6, 3021. doi: 10.21105/joss.03021

[B95] McKinney W (2010). “Data structures for statistical computing in Python,” in Proceedings of the 9th Python in science conference. Eds. van der Walt S. Millman J., 51–56. doi: 10.25080/Majora-92bf1922-00a

[B96] Wickham H. (2016). ggplot2: Elegant graphics for data analysis (New York, Springer-Verlag).

[B97] Wickham H. (2021) Package ‘forcats’. Available at: https://cran.r-project.org/package=forcats.

[B98] Wickham H. Bryan J. (2022) Readxl: Read excel files. Available at: https://github.com/tidyverse/readxl.

[B99] Wickham H. François R. Henry L. Müller K. (2022) Dplyr: A grammar of data manipulation. Available at: https://github.com/tidyverse/dplyr.

[B100] Wiebe K. Robinson S. Cattaneo A. (2019). Climate change, agriculture and food security: impacts and the potential for adaptation and mitigation. Sustain. Food Agric. 2019, 55–74. doi: 10.1016/B978-0-12-812134-4.00004-2

[B101] Wing I. S. De Cian E. Mistry M. N. (2021). Global vulnerability of crop yields to climate change. J. Environ. Econ. Manage. 109, 102462. doi: 10.1016/j.jeem.2021.102462

[B102] Wu T. Song L. Li W. Wang Z. Zhang H. Xin X.. (2014). An overview of bcc climate system model development and application for climate change studies. J. Meteorol. Res. 28, 34–56. doi: 10.1007/s13351-014-3041-7

[B103] Zhang H. Mittal N. Leamy L. J. Barazani O. Song B.-H. (2017). Back into the wild–apply untapped genetic diversity of wild relatives for crop improvement. Evol. Appl. 10, 5–24. doi: 10.1111/eva.12434 28035232 PMC5192947

[B104] Zohary D. Hopf M. Weiss E. (2012). Domestication of plants in the old world: The origin and spread of domesticated plants in southwest Asia, Europe, and the Mediterranean basin (Oxford (UK): Oxford University Press).

