# Supplementary material for: Climate change conditions the selection of rust-resistant candidate wild lentil populations for in situ conservation
Source: Front Plant Sci. 2022 Nov 3;13:1010799. doi: 10.3389/fpls.2022.1010799 (PMC9669080; doi:10.3389/fpls.2022.1010799)
Supplement: Supplementary file 4 [file DataSheet_4.pdf]

Supplementary Material

1 SUPPLEMENTARY TABLES AND FIGURES

1.1 Figures

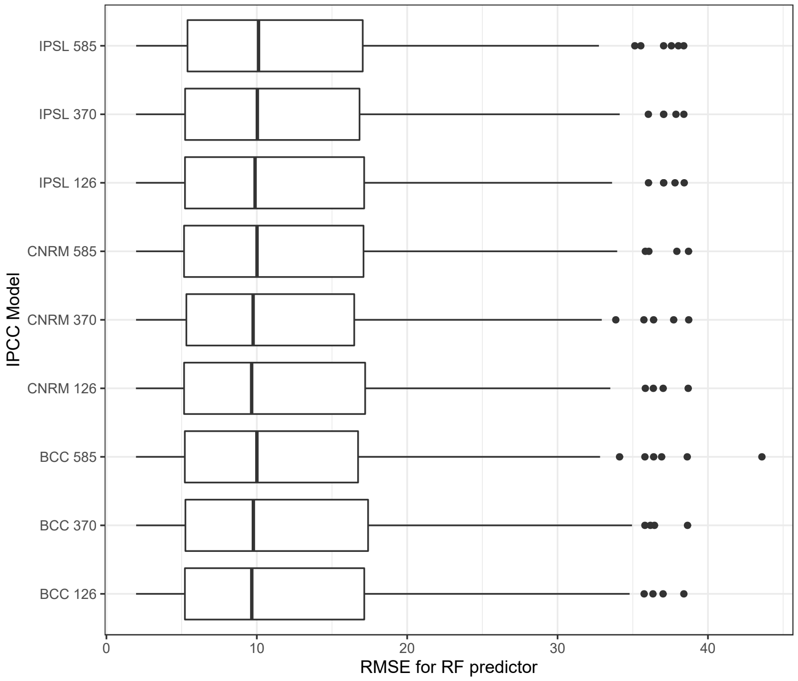

Figure S1. RMSE distributions for 9 IPCC Climate Change models with Random Forest predictor over the calibrated dataset.

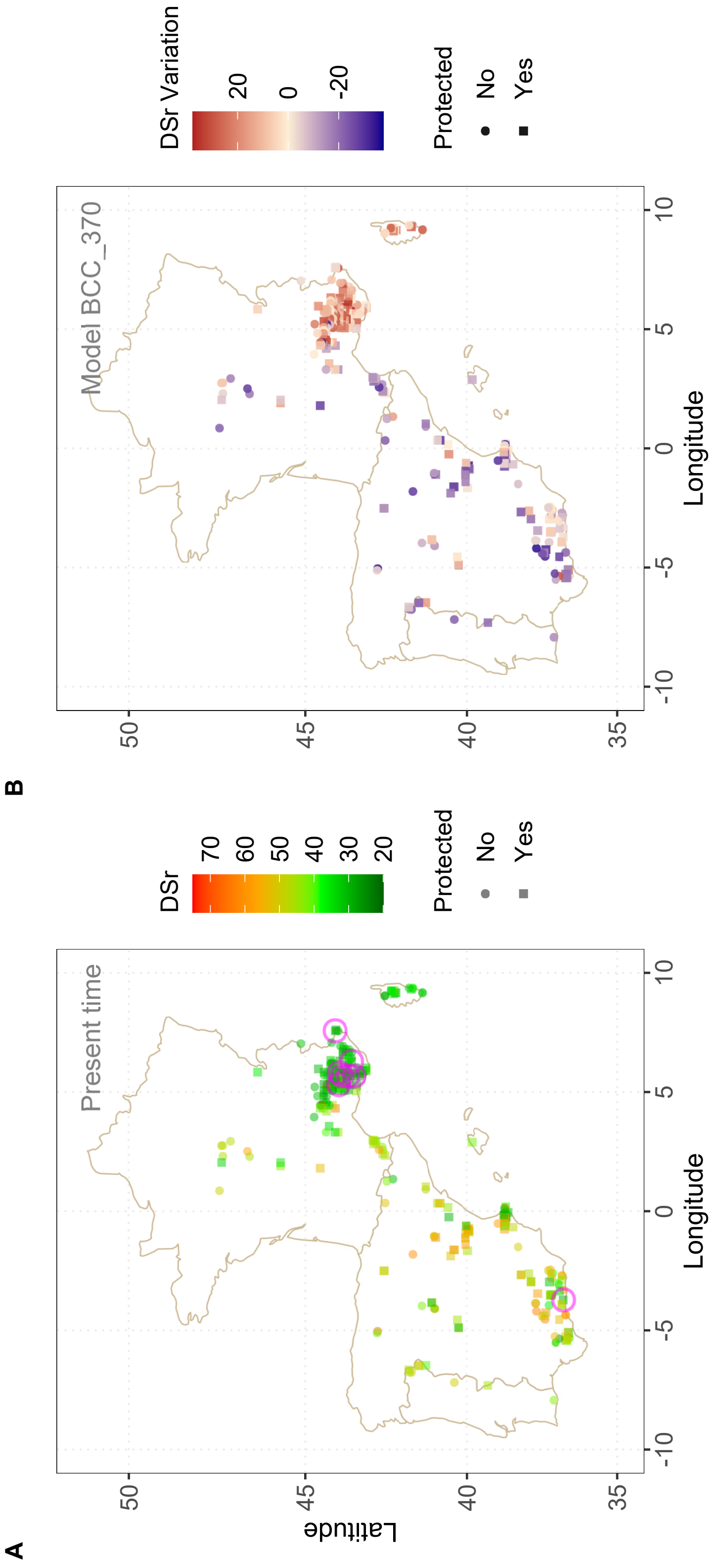

Figure S2. Predicted DSr values for wild populations at present time using Random Forest for the Iberian Peninsula and France (A) and variation under BCC 370 Climate Change Scenario (B)

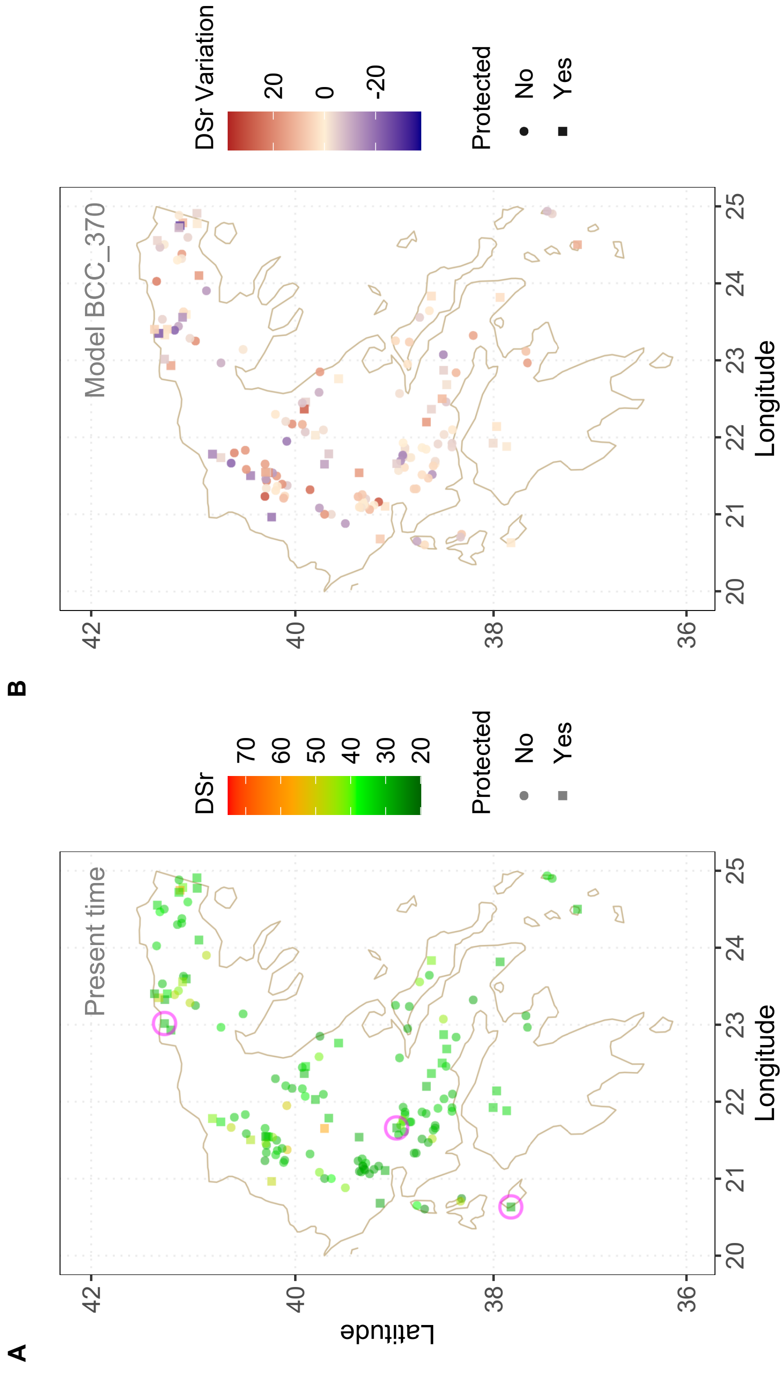

Figure S3. Predicted DSr values for wild populations at present time using Random Forest for continental Greece (A) and variation under BCC 370 Climate Change Scenario (B)

1.2 Tables

Table S1. Summary of sites by Species. Columns Resistan Now, Resistant Future and %High Interest refer to accessions that grow inside protected areas

CWR accessions	All	Protected	Resistant Now	Resistant Future	% High Interest
Lens culinaris subsp. orientalis	7	2	0	0	0.0
Lens ervoides	145	41	14	7	17.1
Lens lamottei	27	16	0	0	0.0
Lens nigricans	404	177	37	9	5.0

Table S2. Median RMSE for IPCC scenarios with RF predictor

Median RMSE	Model
9.52	BCC 126
9.85	BCC 370
9.85	BCC 585
9.69	CNRM 126
9.65	CNRM 370
9.85	CNRM 585
9.84	IPSL 126
9.83	IPSL 370
9.88	IPSL 585

Table S3. Summary of RMSE for BCC 370 scenario

Min.	1st Qu.	Median	Mean	3rd Qu.	Max.	Model
1.90	7.94	13.85	16.48	21.89	57.26	RF
1.16	6.18	12.45	16.25	22.01	81.50	Ridge
4.91	9.99	15.84	18.66	24.44	64.92	XGB
